# Asymmetric Inheritance of Cell Fate Determinants: Focus on RNA

**DOI:** 10.3390/ncrna5020038

**Published:** 2019-05-09

**Authors:** Yelyzaveta Shlyakhtina, Katherine L. Moran, Maximiliano M. Portal

**Affiliations:** Cell Plasticity & Epigenetics Lab, Cancer Research UK–Manchester Institute, The University of Manchester, SK10 4TG Manchester, UK; lisa.shlyakhtina@manchester.ac.uk (Y.S.); katherine.moran@postgrad.manchester.ac.uk (K.L.M.)

**Keywords:** asymmetric inheritance, heterogeneity, plasticity, RNA

## Abstract

During the last decade, and mainly primed by major developments in high-throughput sequencing technologies, the catalogue of RNA molecules harbouring regulatory functions has increased at a steady pace. Current evidence indicates that hundreds of mammalian RNAs have regulatory roles at several levels, including transcription, translation/post-translation, chromatin structure, and nuclear architecture, thus suggesting that RNA molecules are indeed mighty controllers in the flow of biological information. Therefore, it is logical to suggest that there must exist a series of molecular systems that safeguard the faithful inheritance of RNA content throughout cell division and that those mechanisms must be tightly controlled to ensure the successful segregation of key molecules to the progeny. Interestingly, whilst a handful of integral components of mammalian cells seem to follow a general pattern of asymmetric inheritance throughout division, the fate of RNA molecules largely remains a mystery. Herein, we will discuss current concepts of asymmetric inheritance in a wide range of systems, including prions, proteins, and finally RNA molecules, to assess overall the biological impact of RNA inheritance in cellular plasticity and evolutionary fitness.

## 1. Introduction

Decades ago, the fundamental principles that govern DNA folding and DNA-based inheritance were proposed, and with their advent a scientific revolution began. The molecular biology field pushed the boundaries of biological experimentation and thus the principles of genetic engineering were established. Importantly, a vast body of research was directed towards understanding the mechanisms underlying genetic inheritance, therefore uncovering the complexity of cell division and unravelling the multitude of systems controlling the faithful inheritance of DNA copies across cellular generations [1,2]. Many of the mechanisms involved in this process have now been thoroughly described and our current understanding indicates that the improper inheritance of genetic material can promote various disease states [3]. Remarkably, although DNA molecules are segregated to the progeny in a symmetric fashion, many of the integral components of the cell are segregated in an asymmetric manner. An extreme example of the latter can be observed during stem cell division, where it is thought that the asymmetry generated by protein gradients polarizes cellular components, giving directionality to cell division, and generating daughter cells harbouring a rewired regulatory network [4,5,6,7]. This process ultimately generates two genetically identical, but inherently different cells, the original stem cell and its differentiated counterpart that inherits a radically different intracellular environment. Interestingly, the asymmetric inheritance of cellular components seems to be the rule rather than the exception, as many cellular structures also segregate asymmetrically. For instance, it has been acknowledged for decades that mitochondria, whose replication is entirely uncoupled from cell cycle progression, are segregated in a random fashion [8,9]. This asymmetry bears important consequences, as cells that inherit a large number of mitochondria will be able to satisfy all their energetic needs, whereas cells that inherit a reduced number will invariably be constrained until a threshold number of mitochondria is reached. This may indirectly alter the timings of future cell divisions, consequently introducing heterogeneity within a population of cells.

**Figure 1:**
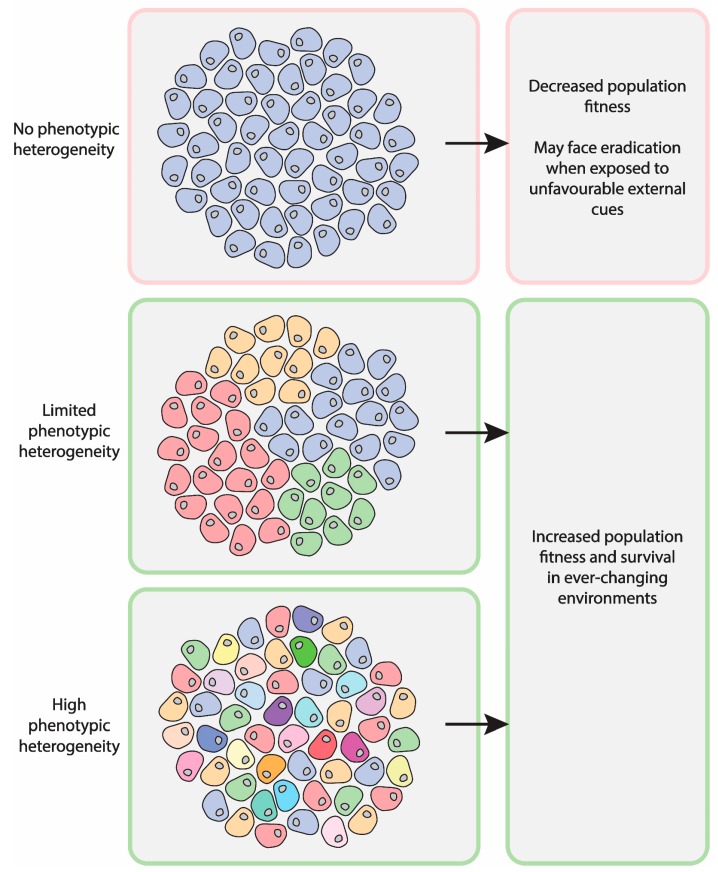
Phenotypic heterogeneity in populations of genetically identical cells. Isogenic populations of cells may display different levels of non-genetic heterogeneity. These differences may result in distinct levels of population fitness and survival capacity when exposed to harsh environmental conditions.

As a large number of biological structures seem to split asymmetrically during cell division, we argue that this biological asymmetry is at the basis of cell plasticity and, as such, it bears a profound biological relevance. Indeed, such a mechanism would generate a panoply of alternative cellular states with minimal energetic expense, all compatible with life, but fundamentally different. All of these potential metastable states could contribute to rapid adaptation to changing environments and thus have key roles in evolutionary fitness (Figure 1). In the upcoming sections, we will explore this concept, discussing hallmark findings with the aim to gradually build a logical framework that supports our hypotheses. 

## 2. Relevance of Asymmetric Segregation

When a cell divides, its contents are split between the two daughter cells produced. In the time leading up to division, many cellular components are duplicated to ensure that daughter cells will inherit everything that is required for them to be viable. However, sometimes this segregation is not equal either via an active mechanism or as a result of stochastic processes, thus producing asymmetry [7,10]. Many factors can influence the downstream effects of asymmetry and whether the differences produced after division will have any functional impact on cell behaviour. In addition to unicellular organisms, such as bacteria and yeast, asymmetric segregation of cellular factors has also been reported to play a role in the development and homeostasis of many multicellular organisms, suggesting it confers a significant fitness advantage [11,12,13,14]. It is argued that the resulting asymmetry generated after cell division is a significant source of heterogeneity within a population of cells in both unicellular and multicellular organisms [4,13]. Interestingly, the asymmetric segregation of deleterious cellular components or waste products into only one daughter cell may also be advantageous, especially in unicellular organisms [15]. If these products were shared into both cells during division, the population may be at risk of dying out unless there was a mechanism which could remove all waste efficiently, which could be costly. Therefore, in addition to segregation of functional components, there is also evidence to support the idea that this process can act to segregate factors which may be deleterious for cellular function.

Importantly, cell fate specification is often facilitated by the asymmetric segregation of factors, which can result in differences between daughter cells that are subsequently propagated to produce functional differences. This idea was first demonstrated in ascidian embryos, where it can be observed that during early cell divisions, the asymmetric segregation of yellow cytoplasm specifies which cells will invariably become muscle [16]. Since then, many more examples of asymmetric segregation have been observed to occur throughout the development of model organisms, such as *Drosophila*, *C. elegans*, and *Xenopus*. Reminiscent of ascidian development, this often involves partitioning of a factor with an ‘all or nothing’ approach, resulting in one daughter cell receiving the full quantity that was present in the original cell and the other daughter receiving none of this particular component [17,18,19]. Although the role of asymmetric segregation was widely accepted as a key feature in the development of *Drosophila, C. elegans*, and *Xenopus*, it was believed for a long time that this phenomenon did not occur during the development of mammals. This was mainly due to the fact that cellular plasticity is recognized as a fundamental feature of mammalian development. For instance, during the early cell divisions, blastomeres appear morphologically equivalent before eventually showing signs of specification at later stages. Consequently, it has been debated as to when the earliest asymmetry between cells appears and even whether they have predetermined fates at these early stages at all. However, a number of recent studies have identified specific molecular differences between blastomeres during mouse development, supporting the idea that asymmetry is present at these earlier stages [20,21]. Heterogeneity between blastomeres has been shown in the expression of a subset of genes, including chromatin modifiers (CARM1) and the targets of pluripotency regulators (Sox21) [20,21]. Thus, a model has been proposed to link these findings, suggesting that variation between blastomeres in the expression of chromatin modifiers will result in differential binding of pluripotency regulators (such as Oct4 and Sox2) [21]. This would therefore result in the heterogeneous expression of lineage specific genes. Indeed, this could function to translate the initial asymmetries present in early stage blastomeres into cell fate decisions over time. Overall, this highlights the conserved role of asymmetric segregation of cellular components during the development of multicellular organisms.

More recently, a distinct role for RNA molecules has been identified even earlier in mammalian development. Indeed, a long non-coding RNA (*LincGET*) is expressed during the two- and four-cell stages and becomes increasingly asymmetric over time [22]. Although it is currently unknown where this asymmetry in *LincGET* expression originates from, it has been suggested that yet to be identified upstream factors which promote *LincGET* differential segregation operate on the system. Alternatively, the authors suggest that these differences could arise from the inherent biological noise present during the earliest stages of blastomere development. Indeed, it has been proposed that small differences between these early cells appear as a result of compartmentalized reactions, which are then amplified over time [23,24]. This would enable asymmetric segregation of factors to gradually drive heterogeneity and fate specification, but still allow plasticity to be maintained at a low level. This ability to proceed with developmental processes and cell differentiation whilst still retaining the capacity to respond to environmental cues and behave plastically appears to be an important concept in mammalian development and is one of the key reasons why it was thought for so long that cells remain equal during these early stages.

Another key example of asymmetric segregation can be observed during stem cell division, which is essential for both development and homeostasis [25]. In essence, the asymmetric nature of stem cell division enables the generation of differentiated cells along with the self-renewal of the anchored stem cell. Often, stem cells reside in particular locations where they produce specialized cell types in a niche-dependent manner [26]. In that context, it has been observed that the niche itself plays an important role in cell type specification as it communicates external signals to the stem cell in order to promote fate decisions. However, in contrast with niche signalling, one of the key mechanisms used to produce two daughter cells with dramatically different fates is the asymmetric segregation of cellular components during the division of the stem cell [4]. In particular, it has been clearly shown that the components inherited by each cell will determine whether it begins the process of differentiation or remains as a stem cell. Overall, the asymmetric segregation of cellular components (including waste products) produces daughter cells with distinct phenotypes leading to the establishment of nongenetic heterogeneity, which enables multiple processes from development and homeostasis to population survival.

## 3. Molecular Fate Determinants

### 3.1. Proteins

A particularly well-studied aspect of asymmetric division is the segregation of intracellular proteins. Proteins, such as transcription factors, play a significant role in cell fate, for example, by conferring stemness or activating the gene expression program required to produce a specific cell type. It follows that these factors are often differentially inherited by daughter cells in order to produce a difference in fate [27]. This mechanism is particularly evident during developmental processes. For instance, during *Drosophila* embryogenesis, the ventral neuroectoderm gives rise to neuroblasts through the process of extrusion. These neuroblasts are polarised along the apical-basal axis, enabling them to undergo asymmetric cell division to produce neurons, after which they become quiescent [28]. This process then repeats during the larval stages to produce neurons in the brain. Although, there are two different types of neuroblasts, the mechanism of asymmetric division is the same. Leading up to cell division, protein determinants are asymmetrically segregated by the action of specific adaptor proteins. These determinants include proteins, such as Numb, Prospero, and Brat, which are found at the basal plasma membrane [29]. The mitotic spindle is then oriented in such a way to ensure that division will result in the asymmetric segregation of protein determinants between the two daughter cells [30]. One cell will remain as a neuroblast, known as self-renewal, and the other will differentiate into a ganglion mother cell, which is able to give rise to neurons.

This model system also demonstrates the consequences of dysregulated asymmetric division. It has been shown that mutations in a number of the determinants and the localisation machinery can lead to the development of tumours [31]. In these mutants, symmetric cell divisions occur and key proteins are not segregated to a single daughter cell, which results in the production of two neuroblasts. Unlike neuroblasts in wild type flies, these “tumour neuroblasts” are unable to exit the cell cycle and continue to proliferate, eventually forming tumour-like structures [31]. There is emerging evidence to suggest a similar process may also underlie certain types of mammalian cancers [32].

### 3.2. Prions

As discussed above, asymmetric segregation plays an important role in the development and homeostasis of multicellular organisms. However, asymmetric cell divisions can also have significant implications for unicellular organisms. It has been known for a long time that cell divisions of budding yeast, *S. cerevisiae*, are asymmetric since the two resulting cells are morphologically distinct. The larger cell is known as the mother and the smaller cell, which buds from the mother, is the daughter. It has been identified more recently that there are also many cellular components which are asymmetrically segregated between mother and daughter cells [14]. One example is prions, which are self-propagating protein isoforms that are thought to act as epigenetic elements in yeast [33,34]. Inheritance occurs via the cytoplasm and therefore there is potential for asymmetry to be produced during cell division, either through active mechanisms or stochastic processes. Some prions are pathogenic, however, some may enhance fitness especially in fluctuating environments, since they are thought to modulate cellular processes and lead to an increase in phenotypic diversity [35].

It has been shown that following stress, prions are retained in mother cells upon subsequent cell divisions. Initially, it was suggested that this was due to the size of prion aggregates increasing after heat shock, however, data now supports a model in which prion aggregates are actively retained in mother cells following stress [36]. This could potentially serve as a mechanism to maintain population fitness by protecting daughter cells from damaged protein aggregates. Thus, the asymmetric pattern of inheritance that prions display represents an exquisite example of the asymmetric segregation of fitness factors through cell division and highlights the relevance of this concept in cellular plasticity.

### 3.3. DNA

Although it is widely accepted that throughout cell division all chromosomes are replicated and further inherited in a single copy per cell, this may not be entirely accurate. Recent compelling evidence, obtained from both unicellular and multicellular organisms, suggests that there can be non-random segregation of chromatids during cell division [37,38]. This asymmetry appears to correlate with the age of the DNA strand, defining DNA age as to whether it was the template strand during replication or the newly synthesised strand. Notably, it has been reported that during division of stem cells, template strands are often retained in the self-renewing cell [39]. This asymmetry in DNA inheritance could confer a fitness advantage to the self-renewing stem cell by minimising the risk of accumulating sequence errors [40]. This model, commonly referred to as the immortal strand hypothesis, is currently being re-evaluated and numerous arguments for and against have already been proposed [39,41,42].

Interestingly, in addition to DNA segregation generating asymmetry during normal cell division, a large body of evidence obtained from the study of pathological states suggests that the asymmetric inheritance of DNA molecules carries profound biological consequences. In that regard, it has been observed that failure to repair double-strand breaks after extensive DNA damage and/or the presence of defective mitosis-related pathways within the cell (kinetochore failure, dysfunctional spindle, etc.) may result in abnormal chromosome segregation that may lead to the formation of extra nuclear DNA structures known as micronuclei. Notably, recent studies have shown that in most cases, micronuclei are inherited by one of the daughter cells, where they can remain intact for several generations or even can get “retaken” back into the nuclei [43,44]. The latter can potentially lead to complex chromosomal rearrangements and may represent a mutagenesis pathway that contributes to the development of different pathological states, such as cancer and congenital disorders [45]. Similarly, extra chromosomal DNA structures can also be formed as a result of homologous recombination. In that context, due to the nature and position of the target locus, homologous recombination can lead to the formation of extra-chromosomal DNA arrangements in the form of circular DNA (extra-chromosomal DNA circles). This phenomenon has been reported for all eukaryotes tested so far, including humans [46,47]. It is particularly relevant in budding yeast, where extra-chromosomal arrangements built up from extra-chromosomal ribosomal DNA circles (ERCs) are asymmetrically inherited by the mother cell during cell division and contribute to ageing. Mechanistically speaking, ERCs occasionally appear in the mother nuclei as a result of homologous recombination in the ribosomal RNA locus and accumulate over time given their ability to self-replicate during the S phase. Recently, it has been shown that ERCs remain anchored to the nuclear pore complex and are retained exclusively by the mother cell via an active mechanism [48,49]. This pathway ensures that the mother cell retains ageing factors, such as ERCs, thus giving rise to heterogeneity within the population and protecting the daughter cell from being born old.

### 3.4. DNA Modifications and Histone Inheritance

All the genetic information that we know about life on Earth is encoded in a rather simple 4-base DNA alphabet—A, G, C, T. However, it has been observed in nature that a subset of DNA bases exist as covalently modified versions of the 4-base code, thus expanding the alphabet information content. Notably, whilst DNA modifications do not lead to any changes in the nucleotide sequence itself, they may affect chromatin structure that in turn can lead to changes in gene expression profiles [50,51]. Importantly, DNA modifications can be stably propagated during mitosis, thus representing a mechanism by which non-genetic information can be stably passed from one generation to another [52].

Across organisms that bear DNA modifications (viruses, bacteria, plants, some fungi, nematodes, vertebrates), the most widespread mechanism is the addition of methyl groups onto cytosine and adenine, leading to modifications, such as 5mC (5-methylcytosine), N4mC (N4-methylcytosine), and 6mA (6-methyladenine) [53,54,55,56]. Although methylation is the most common modification, it has been recently reported that cytosine can also acquire other covalent marks, resulting in 5hmC (5-hydroxymethylcytosine), 5fC (5-formylcytosine), and 5caC (5-carboxylcytosine) [57,58,59,60]. Notably, distinct DNA modifications are differentially represented throughout the evolutionary tree. For example, N4mC was found only in bacteria, 6mA occurs in prokaryotes and some metazoan species, whilst 5hmC, 5fC, and 5caC were all detected in vertebrates, some fungi, and protozoans [55,56,57,58,59,60]. Importantly, 5mC—a cytosine displaying a methyl group onto its fifth carbon—is the most common DNA modification found in vertebrate, fungi, and plant genomes. In particular, it can be found scattered throughout the genome and plays an important role in the regulation of gene expression, including silencing and transcriptional activation. Notably, 5mC occurs mainly in CpG islands and more rarely in non-CpG regions. Indeed, cytosine methylation in CpG islands occurs in a symmetric manner on both DNA strands. Therefore, both parental DNA strands serve as a template to recapitulate the methylation pattern on the daughter strands after replication. Conversely, 5mC in non-CpG islands follows an asymmetric pattern [61,62], suggesting that during replication, only one strand will retain specific DNA modifications that will be inherited by one daughter cell, whereas the other daughter cell will inherit a differentially modified strand. Consequently, two newly generated cells may exhibit distinct gene expression programs that could help to shape cell fate decisions and lead to the establishment of non-genetic heterogeneity within a population of genetically identical cells. Indeed, it has been demonstrated that dynamic modulation of DNA methylation takes place during lineage specific differentiation of haematopoietic stem cells, suggesting that DNA methylation may indeed serve as a mechanism to direct cell fate specification [63,64,65,66]. 

Importantly, 5mC is driven by enzymes known as DNA methyltransferases—a family of cytosine methylases with highly conserved catalytic motifs [54,67,68]. The human genome contains five genes encoding DNA methyltransferases (DNMTs), namely DNMT1, DNMT2, DNMT3A, DNMT3B, and DNMT3L. Whilst DNMT2 and DNMT3L are known as non-canonical family members and do not display enzymatic activity, the other three members are described as canonical methyltransferases and catalyse the covalent binding of a methyl group onto genomic DNA. In that regard, DNMT1 is known as a “maintenance” DNMT which localizes to the replication fork where it binds to the newly synthesized DNA strand to precisely mimic the methylation pattern of the parental strand, which ensures the faithful propagation of methylation marks during cell divisions [69]. In contrast, DNMT3A and DNMT3B are known as “de novo” DNMTs that establish new DNA methylation patterns [69]. Therefore, it is possible to hypothesize that these DNMTs may differentially modify daughter strands after replication. Given the inherent DNA methylation asymmetry and the fact that DNA methylation underlies gene expression programs, it is clear that its asymmetric inheritance may result in two daughter cells exhibiting different fates. 

At another complexity level, the eukaryote genome is “wrapped” around histone octamers to attain a high-order chromatin structure inside the nucleus. The octamers are composed of four histone proteins termed H3, H4, H2a, and H2b, which can undergo various post-translational modifications (methylation, acetylation, ubiquitination, phosphorylation, deamination, ADP ribosylation, sumoylation, histone tail clipping, histone proline isomerization, β-N-acetylglycosamine). These covalent modifications play a crucial role in DNA compaction and accessibility, which directly affects the chromatin landscape and leads to changes in gene expression [70]. For example, histone acetylation and phosphorylation lead to a less compact chromatin structure that presumably makes DNA more accessible to the transcriptional machinery [71,72]. Moreover, histone modifications are essential for other processes, such as DNA repair, replication, and recombination [54]. Importantly, besides the ability of histone modifications to directly influence chromatin structure, they can also bind various chromatin-associated factors that lead to chromatin remodelling and DNA methylation. Although little is known regarding the molecular basis underlying the interaction between histone modifications and the DNA methylation machinery, it has been reported that specific histone modifications can either promote [73,74,75,76,77] or prevent DNA methylation [73,78] by recruiting or impairing the binding of DNMTs. 

Given the fact that chromatin structure plays a fundamental role in gene expression program control, an important question relates to how histones and histone modifications are propagated during mitosis to ensure the inheritance of specific epigenetic programs. During DNA replication, chromatin is disrupted and thus nucleosomes must be disassembled/displaced and histones must be removed from the DNA to allow the progression of the replication fork [79]. It has been suggested that the re-assembly of nucleosomes after replication occurs as a result of two main mechanisms: The recycling of “old” histones that contain post-translational modifications and the incorporation of “new” ones. Indeed, two models explaining the potential molecular mechanism underlying the incorporation of “old” histones after DNA replication has been proposed: The conservative and the dispersive model [80]. Both models suggest that H3-H4 tetramers are recycled and inherited as an intact unit. However, the conservative model proposes that H3-H4 tetramers display a preference in their incorporation onto the leading or lagging strand throughout DNA replication, whilst, on the contrary, the dispersive model hypothesizes that both parental strands randomly inherit H3-H4 tetramers. Notably, these models are not mutually exclusive and may coexist in different systems and at different genomic loci. 

Following those lines, it has recently been reported that H3 histones are asymmetrically segregated during male germline stem cells (GSC) divisions in *Drosophila* [80]. Indeed, it was found that pre-existing “old” H3 histones are inherited by the cell that retains self-renewal potential (the GSC), whereas “new” H3 histones are deposited during DNA replication and are segregated into the daughter cell that undergoes differentiation [81]. Notably, H3 histones do not undergo asymmetric inheritance in symmetrically dividing progenitor cells, suggesting that this phenomenon may be restricted to cells that undergo asymmetric divisions, thus giving rise to daughter cells with distinct fates. Interestingly, it has been recently reported that the Haspin kinase specifically phosphorylates the threonine 3 of “old” H3 histones and directs their asymmetric segregation into GSCs during asymmetric cell division [82]. Notably, as previously discussed, histones bear specific posttranslational modifications that can either promote or silence gene expression, resulting in the establishment of particular gene expression programs. Thus, it has been proposed that pre-existing “old” histones retain specific modifications that enable the inheritance of stem cell fate, whilst the newly synthesized histones segregated into the differentiating cell do not contain stem cell-specific modifications. This would enable the acquisition of a distinct cell fate program that leads to differentiation [80,83]. Along those lines, it has been shown that transcription activating histone modifications (H3 and H4 hyperacetylation and H3 (Lys-4) tri- and di-methylation), as well as transcriptional repressive histone marks (H3 (Lys-9) tri-methylation), are maintained in mitotic cells [84,85,86]. Therefore, histone modifications may indeed serve as a molecular memory device that recapitulates gene expression programs after mitosis in stem cells.

### 3.5. Organelles

In asymmetrically dividing cells, organelles are often segregated in a specific manner during cell division, thus promoting the establishment of distinct phenotypes in daughter cells. For example, in budding yeast, the number of mitochondria inherited by the bud during cell division is tightly controlled and remains constant, whereas the number of mitochondria that are retained in the mother cell diminishes with every division. Interestingly, it has been suggested that mother cells accumulate aged mitochondria, while daughter cells inherit mainly highly functional organelles [87,88]. Notably, asymmetric segregation of mitochondria is not restricted to yeast and is also observed during meiotic divisions of mammalian oocytes. In this particular system, it has been shown that although mitochondria accumulate around the spindle prior to division, they are not equally partitioned into the oocyte and the polar body. Indeed, it is thought that mitochondria are preferentially inherited by the oocyte as a safeguarding mechanism that ensures a steady source of ATP during early development [8]. Interestingly, recent reports suggest that daughter cells asymmetrically inherit aged mitochondria during the division of human mammary stem-like cells [9]. Indeed, the daughter cell that inherits the majority of old mitochondria undergoes differentiation, whereas the one that receives less aged mitochondria maintains the stem cell phenotype. Notably, this particular mode of segregation of old and young mitochondria plays a pivotal role in maintaining stem cell traits in one of the daughter cells. Although further in vitro and in vivo studies are required to extend our understanding of this phenomenon, asymmetric inheritance of aged mitochondria could offer a potential mechanism to promote phenotypic heterogeneity and determine the cell fate of progeny after stem cell division.

Similar to mitochondria, peroxisomes (small membrane-bound organelles containing a large variety of enzymes related to metabolic and non-metabolic processes) also replicate prior to cell division. These structures are subsequently segregated into daughter cells and therefore have the potential to be inherited either symmetrically or asymmetrically. In both mammalian and yeast cells, it is known that peroxisome dynamics, including their segregation at mitosis, are closely linked with the cytoskeleton [89,90]. For instance, in mammalian epidermal stem cells, it has been shown that loss of a peroxisome membrane protein (PEX11b) prevents peroxisome localisation at the spindle poles and leads to their improper segregation upon division [91]. Interestingly, this also results in failed differentiation of daughter cells, highlighting a key role for organelle inheritance in maintaining the balance between proliferation and differentiation. In sharp contrast, yeast peroxisomes are thought to be involved in cellular ageing. Yeast contain a heterogeneous population of both young and old peroxisomes, which are differentially inherited by daughter buds [92]. Old, potentially damaged, peroxisomes are retained in the mother cell, whereas young peroxisomes are actively transported into buds via the actin network. This segregation pattern is maintained over many budding events. In a similar way to prions, this is thought to protect new cells from potentially deleterious organelles, enhancing the overall population fitness.

Many organelles are crucial for cell viability, and so total asymmetric segregation would result in cell death for the daughter cell which received none. Therefore, these components are often inherited symmetrically in terms of quantity, but their quality or functional properties may differ between daughter cells and hence show asymmetric segregation. A key example of this situation is illustrated by centrosomes. Cells contain one centrosome composed of a pair of centrioles. Prior to cell division, these centrioles separate and a new centriole is synthesised beside each [93,94]. During mitosis, each daughter cell inherits one of these new pairs consisting of one parental and one newly synthesised centriole. This process repeats during each cell division. Therefore, in the moments before division, a cell will possess centrosomes containing centrioles of different ages. Consequently, the centrosomes segregated into daughter cells are always inherently different, meaning that the inheritance of centrosomes is always asymmetric. Sometimes this pattern is random, and there is no link between the daughter cell fate or identity and the age of the centrosome. However, in many cells, the direction of this asymmetry is non-random. This has been shown for a number of cell types in different organisms, however, this phenomenon is especially prevalent during stem cell division [95]. As mentioned earlier, *Drosophila* neuroblasts divide asymmetrically to give rise to daughter cells with different fates. One cell will remain a neuroblast whereas the other will become a ganglion mother cell, dividing once or twice before eventually undergoing differentiation. Considering the data from a number of studies detailing the pattern of asymmetric inheritance of aged components in stem cells, it was thought that the neuroblast would retain the older centrosome. However, it was later shown that instead the younger centrosome is inherited by the self-renewing cell, whilst the older centrosome segregates into the cell destined for differentiation [96,97]. Similar to *Drosophila* neuroblasts, mouse neural stem cells also show asymmetric centrosome inheritance. However, in contrast to the situation in Drosophila, these stem cells retain the older centrosome, suggesting that the mechanism or function of this inheritance pattern is not highly conserved between species [98]. In addition to this, centrosomes are also crucial in coordinating the asymmetric segregation of other molecules, which will be discussed later in the review.

Another organelle which is critical for cell viability is the endoplasmic reticulum (ER). Thus, in the same way as it happens with centrosomes, total asymmetric segregation cannot occur. However, asymmetry can be produced in the daughter cells in alternative ways. For instance, in budding yeast, stress conditions lead to the mother retaining all cortical ER whilst the bud receives none [99]. An ER surveillance pathway was proposed to control this segregation and it is thought that this mechanism ensures survival of the mother cell. If stressed ER was segregated equally between the mother and bud, the level of ER function in both cells may drop below what is required for their survival, whereas if all stressed ER is retained in the mother cell, it is likely that this cell can eventually recover and only the daughter will be inviable. Interestingly, this study also found a distinction between the segregation of different subpopulations of ER. In that regard, budding yeast contain both cortical and perinuclear ER, and whilst cortical ER is retained in the mother during stress, perinuclear ER appears to be segregated with the nucleus into the bud as it would be under physiological conditions. It had been previously shown that ER stress is not inherited by daughter cells, prompting the authors to hypothesise that ER stress may be partitioned to the cortical ER, enabling perinuclear ER to be inherited stress-free.

There are many other examples of asymmetric inheritance of the ER in asymmetrically dividing cells [100,101,102]. However, a recent report presented evidence of this process occurring in otherwise symmetrically dividing cells [103]. This study focusses on a specific population of epithelial cells in the *Drosophila* embryo, just prior to these cells becoming neuroblasts. During a specific mitotic event, at this time, it was shown that the ER is asymmetrically inherited, with one daughter receiving a larger quantity than the other. Interestingly, knockdown of an ER membrane protein, Jagunal, prevents this asymmetric segregation. As mentioned previously, neuroblasts undergo an important asymmetric division involving polarisation of a number of cellular components and fate determinants, in addition to spindle orientation, enabling proper segregation. Jagunal mutants which fail to segregate ER asymmetrically also show spindle orientation defects at this later time point. Therefore, this data suggests that the asymmetric segregation of the ER could be important for the proper asymmetric division of neuroblasts, and highlights the role that asymmetric segregation of organelles can play in cell fate specification.

## 4. Differential RNA Segregation and Inheritance

The first visual evidence of the asymmetric localization of specific mRNA molecules was provided in the early 1980s when Jeffery and colleagues used RNA in-situ hybridization to demonstrate differential β-actin mRNA distribution in the cytoplasm of ascidian eggs [104]. Since then, numerous studies have provided further support to this phenomenon and demonstrated that asymmetric RNA segregation can provide both spatial and temporal control of protein synthesis [105,106,107,108,109]. Importantly, this process also plays a fundamental role in the establishment of morphogen gradients and the differential distribution of cell fate determinants during asymmetric and symmetric cell divisions. In multicellular organisms, the specific localization of particular RNA molecules is required for neuronal plasticity, stem-cell differentiation, and embryogenesis [110]. The importance of this process is exemplified by the severe developmental defects produced following mis-localization of specific mRNAs during *D. melanogaster* embryogenesis. For instance, failure to localize *oskar* and *nanos* mRNA in the *Drosophila* embryo leads to the development of a second abdomen in the place of the head and thorax [111,112]. In 2007, it was reported that 71% of the mRNAs expressed during *Drosophila* embryogenesis show a clear specific subcellular localization, suggesting that most mRNAs have a particular distribution pattern during development [113]. These and other examples suggest that the subcellular distribution of various RNA molecules and their asymmetric segregation patterns may play a central role in the regulation of core cellular mechanisms.

One of the best-characterized examples of asymmetric segregation of a cell fate determinant is the specific localization observed for *ASH1* mRNA during asexual reproduction in budding yeast [114,115]. During anaphase, *ASH1* mRNA is asymmetrically sorted and concentrated at the cortex of a newly formed bud and then specifically inherited by the daughter cell. This leads to the expression of Ash1p exclusively in the daughter cell, which enables the specific repression of HO endonuclease and prevents the localized recombination in the MAT locus required for mating type switching (from α to a and vice versa). Therefore, this cell loses the capacity to convert one haploid mating type to the other. Notably, the mechanisms of assembly and active transport of *ASH1* mRNA are rather well understood [116]. In the nucleus, *ASH1* mRNA is co-transcriptionally recognized by the RNA binding protein, She2p, followed by association with Loc1p, which stabilizes a complex that is subsequently exported to the cytoplasm. Once in the cytoplasm, Loc1p is replaced by the cytoplasmic RNA binding protein, She3p, leading to the formation of a complex composed of She2p and She3p that specifically interacts with any of the four *ASH1* mRNA cis-acting localization elements [117,118]. She3p directly binds to the C-terminal tail of Myo4p, a molecular motor that enables directional movement along actin filaments to the tip of the daughter cell. Consequently, *ASH1* mRNA is only inherited by the daughter cell, therefore preventing mating type switching. This observation provides clear evidence that RNA molecules can lead to the establishment of phenotypic heterogeneity following asymmetric inheritance.

Asymmetrically localized RNA molecules also play a crucial role in axis formation and primary patterning of *Drosophila* and *Xenopus* embryos [11]. For instance, formation of the anterior-posterior axis in *Drosophila* embryos is controlled by the localization of *gurken (grk)* and *bicoid (bcd),* as well as *oskar (osk)* and *nanos (nos)* mRNAs, to the anterior and posterior pole of the oocyte, respectively [119,120,121,122,123,124,125,126]. Importantly, it has been shown that these RNAs are asymmetrically sorted following two alternative pathways, either via active microtubule-mediated transport or as a result of diffusion and trapping [127,128,129]. In particular, *grk, bcd*, and *osk* mRNAs are sorted to their final destination via microtubule-mediated transport, whereas *nos* mRNA appears to be exclusively partitioned via a diffusion mechanism [127,128,129]. The fact that both mechanisms co-exist and cooperate to ensure the proper segregation of RNA-based fate determinants during early development supports the idea that both sorting pathways are fundamental for the successful development of multicellular organisms.

Intriguingly, in addition to its function in the recruitment of abdominal determinants at the posterior pole, Oskar participates in the formation of membrane–free ribonucleoprotein complexes termed P-granules. These RNA-rich maternally inherited cytoplasmic bodies harbour nearly 200 different RNA molecules and are a hallmark of all germ cells studied so far [130]. Although the presence of P-granules seems to spread widely in the evolutionary tree, the timing of their formation differs between species. For instance, in mammals, germ granules are not detected in oocytes or early embryos and are only formed in primordial germ cells. In contrast, P-granules are continuously present throughout the development of *Drosophila, Xenopus, C. elegans*, and zebrafish [131,132]. Notably, they are transmitted from oocyte to embryo and segregate with the germ lineage, playing an important role in cell fate specification and gonad formation. In that regard, it has been found that asymmetric segregation of P-granules during the first four divisions of *C. elegans* embryogenesis plays a crucial role in the proper development and functioning of gonads in a fully developed organism when subjected to stress conditions, such as high temperatures (24–26 °C) [133]. Briefly, mutant animals that fail to asymmetrically localize P-granules during the first stages of development have normal gonad development and are fertile. However, at restrictive temperatures (24–26 °C), 20% of the animals that fail to segregate the P-granules into the germ lineage lack gametes display underdeveloped gonads and are sterile. This suggests that the asymmetric partitioning of P-granules is not essential for germline formation, but rather serves as a mechanism that increases germline resistance to unfavourable external cues. Importantly, several P-granules components, such as DEPS-1, GLH-1, and PGL-1, have been shown to play a fundamental role in oocyte and sperm production at restrictive temperatures [134,135,136]. Notably, double mutant animals for P-granule components (*ghl-1/ghl-4* or *pgl-1/pgl-3*) are sterile at low (16 °C) and high temperatures (26 °C), suggesting that P-granules are required for *C. elegans* fertility at extreme temperatures. Interestingly, it has been shown that a key P-granule component, DEPS-1, may promote the specific accumulation of particular mRNAs (*glh-1* and *rde-4*) within P-granules [136], thus suggesting that the mRNAs which are asymmetrically inherited as part of the P-granules may contribute to germ cell proliferation and the fertility of an organism.

Notably, it has been suggested that PIWI-interacting small RNAs (piRNAs) also segregate asymmetrically during *C. elegans* embryogenesis as an integral part of P-granules [137,138,139]. In particular, it has been shown that PRG1—an Argonaute protein involved in piRNA biosynthesis—co-localizes with these structures and interacts with 21U-RNA, a subclass of piRNAs which is expressed in the germline of *C. elegans* [140,141,142,143,144]. Indeed, recent data supports the idea that PRG1/21U-RNA complexes play an important role in temperature-dependent germline processes, such as fertility [137]. Mechanistically, PRG1/21U-RNA complexes appear to repress the expression of spermatogenesis-related transcripts in trans through imperfect binding (21U-RNA:mRNA), whilst also eliciting a secondary 22G-siRNA response (RdRP—RNA-dependent RNA polymerase mediated). The latter response amplifies the initial molecular signals to ensure that a specific silencing program is transmitted to the germline, potentially to modulate key developmental pathways [145,146,147]. Interestingly, two other members of the Argonaute family—ALG-3 and ALG-4—are required for the localization of 26G-RNAs (a subgroup of RdRP-dependent small RNAs) to P-granules at the late pachytene stage of male gametogenesis. This suggests that the inheritance of silencing programs is widespread rather than restricted to a single mode of silencing. In this context, as previously suggested as the mechanism of action for 21U-RNA, 26G-RNAs appear to specifically silence spermatogenesis-expressed mRNAs [148]. Overall, P-granules contain multiple cell fate determinants in the form of RNA and protein complexes, and modulate key biochemical processes, such as translation and post-transcriptional silencing, that play crucial roles in ensuring the successful development of the germline under normal and stress conditions.

Although it is now widely accepted that P-granules are key components in the asymmetric segregation of transcriptional/silencing programs, it is important to question how it is possible that these molecular aggregates remain assembled in the cytoplasm. Recent reports suggest that liquid-liquid phase separation may provide a mechanism to organize numerous complex biochemical reactions in space and time [149]. Interestingly, as P-granules exhibit the behaviour of a liquid droplet (fusion, dripping, and wetting), it was therefore suggested that their formation relies on a phase transition process in which soluble proteins and RNA molecules are condensed, resulting in the formation of these non-membrane structures [150,151,152]. Moreover, it has been proposed that the assembly of P-granules could be achieved by multiple weak interactions between RNA-binding proteins and RNA molecules. Notably, RNA-binding proteins usually contain modular structures composed of multiple short repeats that create versatile binding surfaces. Although single modules exert a relatively weak affinity towards their cognate RNAs, the presence of multiple binding sites creates an interaction surface that increases the overall affinity and specificity through co-operativity [153,154,155]. Interestingly, it has been suggested that phase transition is driven by RNA molecules and is likely to be determined by salt concentration and the local molar ratio of specific RNAs and proteins as either too high or too low ratios do not favour phase transition [156]. This was demonstrated in vitro by using an RNA-binding protein, Whi3, that cannot phase separate on its own at low protein and high salt concentrations, but condenses into liquid-like droplets once cyclin mRNA (*CLN3*) is added. Consequently, it was also shown that *CLN3* mRNA promotes phase separation at physiological salt conditions, suggesting that RNAs may indeed play an essential role in promoting phase transition. Importantly, RNA molecules are not simply “passive passengers” in the process of phase transition [156]. Indeed, it has been shown that distinct mRNAs determine the specific biophysical properties of liquid-like droplets. Interestingly, Whi3 exerts a dual function by participating either in nuclear division or establishing polarity depending on whether it is bound to *CLN3* or *BNI1* mRNA, respectively [157,158]. The assembly of Whi3 with either *CLN3* or *BNI1* is driven by the local concentration of Whi3, *CLN3* mRNA, and *BNI1* mRNA. This results in the formation of dynamic liquid-like droplets with different physical properties, such as distinct densities of Whi3 binding sites, the ability to fuse and the viscosity of the droplets [156]. Importantly, mutant Whi3 that cannot bind RNA assembles into filamentous, elongated, salt-resistant structures that may represent potentially toxic fibres [156]. This suggests that the presence of RNA may prevent these solid-phase processes and avoid potential pathological states [159]. Notably, the RNA-interacting protein, Puf2, associates with Whi3/*BNI1*, but not with Whi3/*CLN3*, suggesting that specific RNA molecules within the droplets can determine their composition and thus their biological function. Based on this recent study, the authors hypothesized that RNA molecules not only contain genetic information, but also encode structural and biophysical properties that enables the formation of phase-separated granules [156].

Interestingly, a distinct mechanism of differential RNA segregation was described for early embryonic cleavages of the mollusc embryo [160,161]. In this case, asymmetric mRNA inheritance is achieved by RNA accumulation on a particular interphase centrosome that is inherited by one daughter cell during cell division. In that context, RNA movement to the cortex does not occur unless it was previously loaded onto centrosomes. The entire complex is further transported by actin filaments to the region of the cell cortex that is subsequently inherited by only one daughter cell [160]. Interestingly, the presence of RNA in centrosomes is not restricted to mollusc embryos as it has also been reported for other model organisms [113,162]. For instance, a genome wide study performed by Lecuyer and colleagues revealed 24 transcripts that localize to centrosomes in the *Drosophila* embryo [113]. Importantly, centrosome-mediated asymmetric inheritance is not restricted to cell divisions observed during embryogenesis. It has been reported that during supposedly symmetric mitosis of self-renewing human embryonic stem cells and other mammalian cultured cell lines, proteins targeted for proteasome degradation (pSmad1, phospho-β-catenin, and other ubiquitinated proteins) localize in the centrosome region and are mainly segregated into one daughter cell [163,164]. Interestingly, in higher organisms, the old and young centrosomes show different behaviours and are functionally distinct for part of the cell cycle [165]. Overall, this allows us to hypothesize that different centrosomes might associate with different subsets of RNA and protein molecules that will consequently be differentially inherited during mitotic cell division, thus giving rise to non-genetic diversity.

Another structure that is asymmetrically inherited in various cell types is the midbody, located at the intercellular bridge during cytokinesis [166]. It is formed during the ingression of the cleavage furrow and is composed of compacted central spindle microtubules as well as hundreds of proteins, including kinases and phosphatases, known to play key roles during cell growth and differentiation. Interestingly, it was demonstrated that the composition of the mitotic midbody differs from the one found in post-mitotic cells, suggesting that it undergoes remodelling in the cell that inherits it. Therefore, the asymmetric inheritance of midbody components may lead to profound functional consequences, as each cell may inherit different fate determinants, thus promoting the establishment of non-genetically encoded cellular heterogeneity within the progeny.

## 5. Asymmetric Inheritance of Cell Fate Determinants During Stem Cell Divisions

A universal feature of all stem cells is their ability to self-renew as well as give rise to more differentiated lineage-specific progeny. It is generally accepted that in order to achieve this, two distinct mechanisms coexist: The intrinsic and the extrinsic pathways. The intrinsic model suggests that the regulators promoting self-renewal undergo polarized localization, leading to asymmetric segregation upon cell division and ensuring their inheritance by only one daughter cell [4]. This phenomenon has been observed in the progenitors of the peripheral and central nervous system (sensory organ precursor cells and neuroblasts, respectively) of *Drosophila*. Following two consecutive asymmetric cell divisions, sensory organ precursor (SOP) cells give rise to four different cell types found in external sensory organs (socket, hair, sheath, neuron). It has been shown that the asymmetry in these cell divisions is achieved by the specific segregation of Notch signalling regulators into one daughter cell [167,168,169]. In contrast to SOP cells, neuroblasts generate large cells that are characterized by neuroblast self-renewing properties and smaller ganglion mother cells that are capable of only one more cell division to generate two neurons. The generation of phenotypically distinct daughter cells is a result of specific segregation of multiple proteins (Par-3, Par-6, aPKC, Inscuteable, Pins, GαI, Mud) to the apical side of the neuroblast cortex [5,6,7]. Interestingly, this specific accumulation leads to the localization of cell fate determinants (Numb, Prospero, Brat) to the basal side, which are inherited by the ganglion mother cell [4,170]. It has been evidenced that Numb, Prospero, and Brat play a pivotal role in cell cycle exit and the induction of terminal differentiation. Interestingly, the mediator of Prospero and Brat segregation—Miranda—was also shown to interact with the double-stranded RNA-binding protein, Staufen, that transports *Prospero* mRNA, leading to its asymmetric accumulation [171,172]. Moreover, *Miranda* mRNA also asymmetrically segregates during mitosis, but, contrary to Miranda protein, it localizes at the apical cortex [173]. These findings suggest that, similar to protein molecules, RNAs can also be specifically segregated during stem cell divisions, resulting in phenotypic differences between daughter cells. Indeed, recent studies have shown that the double-stranded RNA-binding protein, Staufen2, together with a subset of mRNA molecules (*Bbs2* and *Trim32*, amongst others) is asymmetrically segregated during neural stem cell division in the developing mouse cortex and plays a critical role in normal cortical development [174]. Staufen2 is mainly inherited by the intermediate progenitor cells (IPCs), which undergo a limited number of cell divisions to generate neurons and glial cells. Interestingly, cargo mRNAs that are asymmetrically inherited with Staufen2 are involved in a number of key processes, such as mitotic exit, mRNA transport, and metabolism of non-coding RNAs [174], thus suggesting that the asymmetric inheritance of RNA molecules could play a crucial role in stem cell differentiation.

Alternatively, the extrinsic model suggests that niche-anchored stem cells have the capacity to establish an axis of polarity during interphase that allows specific positioning of the mitotic spindle perpendicular to the stem cell niche. This ensures that following cell division, only one daughter cell maintains a physical contact with the stem cell niche and thus holds the potential to self-renew, whereas the other daughter cell acquires the ability to differentiate due to the loss of contact with the niche [4]. This suggests that external signals from the microenvironment may play a crucial role in the asymmetric cell division of stem cells that give rise to phenotypically distinct daughter cells. Although this mechanism is best characterized in *Drosophila* and *C. elegans*, it is also observed in other model systems, indicating its widespread nature [4,169,170,175,176]. In that regard, it has been reported that Wnt3a, a ligand that induces the Wnt/β-catenin pathway, drives asymmetric cell division in mouse embryonic stem cells in vitro [176]. Briefly, Wnt3a co-localizes with the Wnt receptor, LRP6, adenomatous polyposis coli (APC) protein, β-catenin as well as the old centrosome [95,176], which are further asymmetrically segregated into the daughter cell that maintains pluripotency. Given that multiple RNA molecules can be loaded onto centrosomes, it is possible to hypothesize that different classes of coding and non-coding RNAs (miRNA, piRNA, lncRNA, etc.) may also be asymmetrically inherited during stem cell divisions, thus potentially driving specific cellular programs leading to either cell differentiation or self-renewal.

## 6. Discussion

Cellular plasticity describes the ability of a cell to alter its state in response to a stimulus, such as a change in the environment or a signal from a neighbouring cell. Within a population of cells, it has been shown that there can be significant variation in both the extent to which cells exhibit plasticity, and the nature of the response to the same stimulus [177,178]. Since this variation can be seen in clonal populations of cells, it suggests that non-genetic components, such as organelles, proteins, and RNA molecules, have the ability to influence the plasticity of an individual cell. It follows, therefore, that asymmetric segregation of these cellular components could lead to differences in the plastic responses of daughter cells. A key example of this is shown by the asymmetric division of stem cells as detailed earlier. In this case, the process of asymmetric division aims to produce one cell which retains stemness and therefore a high level of plasticity, whereas the other daughter cell should lose stemness and plasticity through the process of differentiation. Therefore, the asymmetric inheritance of fate determinants may have a potential to influence the plasticity of daughter cells. This concept highlights the significance of the asymmetric segregation of cellular components, since plasticity (the production of alternative cellular states) is key to both the survival of unicellular organisms and the development and homeostasis of multicellular organisms. Along these lines, it becomes clear that any changes to the segregation patterns of a cellular component could have significant implications for the daughter cells and the organism as a whole, illustrated by the numerous examples given in this review [31,91,103,111,112,133,179].

Notably, a high level of plasticity is thought to provide an adaptive advantage, particularly in changing environments, and so it could be suggested that asymmetric inheritance of a component to maintain plasticity increases fitness and protects a population from eradication by potentially lethal exposures. However, this may not always be the case. If the asymmetric segregation of cellular components influences the plastic response of a cell to a stimulus and the resulting response is not optimal, this process is maladaptive for the cell.

Overall, it is clear that the coordination between asymmetric inheritance and cellular plasticity may result in the establishment of multiple stable phenotypic states that give rise to non-genetic heterogeneity within isogenic populations of cells. One possibility is that this heterogeneity will not be maintained, because of the lack of feedback mechanisms to amplify the differences, or due to mechanisms that may even act to return the daughter cells back to their original cell states. However, if this heterogeneity does persist, there could be many implications for the cells themselves and the population as a whole. Population heterogeneity can in some cases be a valuable contributor to population fitness, especially in unpredictable or fluctuating environments. In a similar way to plasticity, heterogeneity produced by asymmetric inheritance can also offer evolutionary advantages since it can broaden the range of environments that can be tolerated. Indeed, in clonal populations of bacteria, it has been observed in numerous different species that often there exists a small subpopulation of cells with a slow or arrested growth state [180]. In a constant environment, this phenotype appears to have no functional significance. However, when exposed to antibiotics these cells endure and persist, enabling the population to survive the challenge. Importantly, this has been shown to occur without genetic changes that could confer resistance to the toxic agent. Moreover, following treatment, slow proliferating cells are able to spontaneously switch their growth state and produce a new population with the same composition and dynamics as the original one, including cells which are susceptible to the antibiotic. Computational modelling of this process suggests that this stochastic switching occurs at steady state and that the resistant subpopulation of cells is present as a result of pre-existing heterogeneity in the population and not as a plastic response to the drug [181]. This behaviour is known as a bet hedging strategy and it describes a phenomenon in which heterogeneity is produced within a population “in the hope” that at least some cells will be well adapted to whatever changes might occur in the future environment. Notably, this phenomenon is not restricted to bacteria cell populations and has been evidenced to exist in numerous clonal populations of cancer cells [182,183,184]. In that regard, it has been demonstrated that drug tolerant states can emerge in genetically homogeneous populations of cancer cells following treatment with cytotoxic agents. Notably, it has been shown that once the drug treatment is discontinued (known as “drug holiday”), cancer cells revert to their initial response and re-gain sensitivity to the challenging agent. Together, this suggests that the phenomenon of acquired resistance may have a reversible nature and therefore does not necessarily result from the acquisition of stable genetic mutations [182,183]. Notably, reversible resistance to therapeutic paradigms has been reported in clinical settings. For instance, it has been shown that non-small cell lung carcinoma patients display an initial efficient response to EGFR tyrosine kinase inhibitor; however, this therapeutic approach rapidly fails following sustained drug administration. Interestingly, patients that were subjected to a period of “drug holiday” regain sensitivity to this treatment, indicating that the acquisition of a non-genetically based reversible drug tolerant state to antitumor agents is indeed a relevant phenomenon in the clinic [185,186,187,188]. Overall, these observations suggest that cancer cells can harbour non-genetic mechanisms to escape cell death and even proliferate in the presence of a therapeutic paradigm.

**Figure 2:**
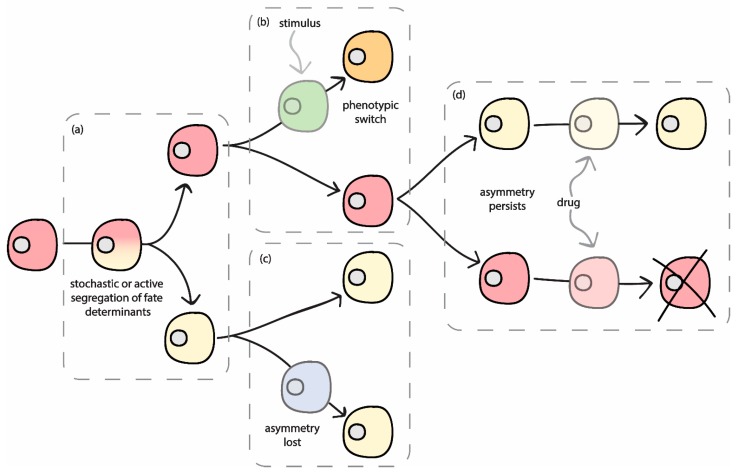
Potential consequences of asymmetric cell division. Phenotypically different cells may arise in a clonal population by passive—diffusion-mediated—or active mechanisms (**a**). Subsequently, this non-genetic heterogeneity may persist in clonal populations and may set the basis for phenotypic switches upon exposure to various external stimuli (**b**). On the other hand, phenotypic differences may be eliminated shortly after cell division, as specific mechanisms might act to diminish cell division-induced asymmetry (**c**). Moreover, phenotypic variation in cell populations may increase fitness and enable survival of some cells under exposure to lethal cues (**d**).

Along those lines, we hypothesize that this non-genetic heterogeneity within a cell population can arise as a result of asymmetric inheritance of cell fate determinants during cell divisions (Figure 2). However, which are the mechanisms that guide asymmetric inheritance of cellular components that may change the cell fate? Is this phenomenon driven by deterministic or stochastic mechanisms? Numerous examples described in this review suggest that asymmetric segregation of different protein and RNA molecules and their specific partitioning into daughter cells can be strictly controlled and coordinated through the development of multicellular organisms and in the process of stem cell differentiation (Table 1). However, the inherent stochasticity or ‘noise’ in biological processes suggests that cellular components are unlikely to be truly homogeneous throughout the cell, even if no dedicated pathways are working to produce and/or maintain this asymmetry. Thus, there is a chance that asymmetric inheritance will occur during cell division simply as a result of stochasticity in the local concentration of cellular components. This heterogeneity may be influenced by a number of factors, including diffusion rates, localised reactions, and interactions between molecules. It seems logical that asymmetric segregation as a result of inherent intracellular heterogeneity could have played a role in the very ancient history of cellular evolution. Perhaps inherent asymmetry provided the basis for the evolution of more active segregation mechanisms detailed earlier in this review. It could even be postulated that these more ‘deliberate’ forms of asymmetric inheritance evolved alongside multicellularity when a requirement for increased regulation and complexity during development arose.

In summary, genetically identical cells within a population of somatic cells that undergo supposedly symmetric mitosis are often characterized by phenotypic heterogeneity that is a result of numerous non-genetic mechanisms operating within the cell and population in general (Figure 2). 

Non-genetic heterogeneity can be driven by stochastic gene expression that varies between the cells of the population and within a single cell over time, metabolic communication among the cells of the population, influence of the microenvironment, and asymmetric inheritance of cell fate determinants, such as proteins, biochemical reactions, signalling and metabolic pathways, and RNA molecules. The co-existence of all these processes may suggest that the behaviour of the cell population is rather stochastic and random. However, this does not appear to be the case. The variable response of clonal populations to specific external cues is often highly reproducible and thus cannot be explained by solely stochastic mechanisms. It could be suggested, therefore, that phenotypic heterogeneity has some boundaries and is ordered to a certain extent. These limits ensure that the population does not lose its identity, but also allows the co-existence of multiple stable states that may have an important adaptive potential in an ever-changing environment (Figure 1).

How is this order achieved in such noisy, messy systems? How many phenotypic states can co-exist within a clonal population of cells? Which are the factors and mechanisms that generate and maintain a certain level of non-genetic heterogeneity? Are these stable states functionally important for the fitness of the population and its adaptive evolution? Answers to these questions will help to better understand the complex phenomenon of phenotypic heterogeneity and its role in evolutionary processes.

## Figures and Tables

**Table 1:** Fate determinants asymmetrically inherited during cell divisions.

Nature of Cell Fate Determinant	ID	Function	Reference
**mRNA**	*ASH1*	Asymmetrically segregates into daughter cell and prevents mating type switching in budding yeast	[114,115,116,117,118]
	*Gurken (grk),* *Bicoid (bcd)*	Asymmetrically localizes to the anterior pole of oocytes and early embryos. Essential for axis formation and primary patterning in *Drosophila* and *C. elegans*	[119,123,126,128]
	*Oskar (osk),* *Nanos (nos)*	Asymmetrically localizes to the posterior pole of oocytes and early embryos. Essential for axis formation and primary patterning in *Drosophila*	[112,120,124,125,127]
	*BNI1*	Potential crucial role in P-granules formation through phase separation. Participates in the establishment of polarity	[156]
	*Prospero*	Asymmetrically inherited during stem cell differentiation	[171,172]
	*Glh-1, rde-4*	Contributes to germ cell proliferation and fertility in *C. elegans*	[135]
**Non-coding RNA**	21U-RNA	Co-localizes with the P-granules that are asymmetrically segregated into the germ line of *C. elegans.* They may promote specific silencing programs	[140,141,142,143,144,145,146,147]
	26G-RNA	Co-localizes with the P-granules at late pachytene stage of male gametogenesis in *C. elegans.* They may promote specific silencing programs	[148]
**Proteins**	Numb, Prospero, Brat	Asymmetrically inherited during stem cell differentiation	[29]
	Par-3, Par-6, aPKC, Inscuteable, Pins, GαI, Mud	Asymmetrically inherited during stem cell differentiation	[4]
	DEPS-1, GLH-1, PGL-1	Participate in oocyte and sperm production at restrictive temperatures	[134,135,136]
	PRG1	Participates in temperature-dependent germline processes, such as fertility in *C. elegans*	[137,140,141,142,143,144]
	ALG-3, ALG-4	Required for the localization of 26G-RNAs to P-granules at late pachytene stage of male gametogenesis	[148]
	H3 histone	Asymmetrically inherited during male germline stem cell divisions in *Drosophila*	[81,82]
**Prions**	[PSI1+]	Increased nonsense suppression	[34]
**DNA**	Extra-chromosomal DNA circles	Yeast ageing	[48,49]
	Micronuclei	Potential mutagenesis pathway that results in pathological states (e.g., cancer)	[43,44,45]
**Organelles**	Mitochondria	Yeast ageing Asymmetrically inherited during stem cell differentiation	[8,87,88]
	Peroxisomes	Asymmetric segregation in mammalian epidermal stem cells required for differentiation of daughters. Also contribute to cellular ageing in yeast	[91,92]
	Centrosomes	Asymmetric inheritance in stem cells. Also required for asymmetric segregation of protein and RNA molecules	[96,97,98,113,160,163,164]
	Endoplasmic reticulum	Segregated to mother cell upon stress in yeast to promote survival. Asymmetric inheritance during *Drosophila* embryogenesis required for proper neuroblast division.	[99,103]
